# Ultrasound Plane Pose Regression: Assessing Generalized Pose Coordinates in the Fetal Brain

**DOI:** 10.1109/TMRB.2023.3328638

**Published:** 2023-10-31

**Authors:** Chiara Di Vece, Maela Le Lous, Brian Dromey, Francisco Vasconcelos, Anna L. David, Donald Peebles, Danail Stoyanov

**Affiliations:** EPSRC Center for Interventional and Surgical Sciences and the Department of Computer Science, University College London, WC1E 6DB London, U.K.; WEISS, Elizabeth Garrett Anderson Institute for Women’s Health, and the NIHR University College London Hospitals Biomedical Research Center, University College London, WC1E 6DB London, U.K.; EPSRC Center for Interventional and Surgical Sciences and the Department of Computer Science, University College London, WC1E 6DB London, U.K.; WEISS, Elizabeth Garrett Anderson Institute for Women’s Health, and the NIHR University College London Hospitals Biomedical Research Center, University College London, WC1E 6DB London, U.K.; EPSRC Center for Interventional and Surgical Sciences and the Department of Computer Science, University College London, WC1E 6DB London, U.K.

**Keywords:** Fetal ultrasound, convolutional neural network, plane localization, alignment, fetal brain.

## Abstract

In obstetric ultrasound (US) scanning, the learner’s ability to mentally build a three-dimensional (3D) map of the fetus from a two-dimensional (2D) US image represents a significant challenge in skill acquisition. We aim to build a US plane localization system for 3D visualization, training, and guidance without integrating additional sensors. This work builds on top of our previous work, which predicts the six-dimensional (6D) pose of arbitrarily oriented US planes slicing the fetal brain with respect to a normalized reference frame using a convolutional neural network (CNN) regression network. Here, we analyze in detail the assumptions of the normalized fetal brain reference frame and quantify its accuracy with respect to the acquisition of transventricular (TV) standard plane (SP) for fetal biometry. We investigate the impact of registration quality in the training and testing data and its subsequent effect on trained models. Finally, we introduce data augmentations and larger training sets that improve the results of our previous work, achieving median errors of 2.97 *mm* and 6.63° for translation and rotation, respectively.

## I Introduction

**Fig. 1:**
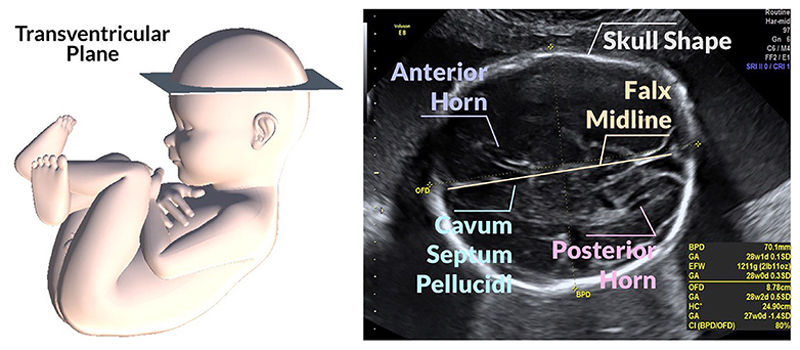
TV SP to evaluate fetal biometry in the brain. This plane needs to show the skull shape, the CSP, the posterior horn of the lower lateral ventricle, the anterior horns of the lateral ventricles, the skull shape, and the falx midline.

**F**ETAL US is a non-invasive, real-time, and cost-effective diagnostic tool for monitoring fetal growth and anatomy throughout gestation [1]. During routine mid-trimester fetal US scan, the sonographer acquires the SP, predefined anatomical planes defined by scientific committees to promote international guidelines for fetal US images [2]. Specifically, the TV SP needs to show the skull shape, the cavum septum pellucidum (CSP), the posterior horn of the lower lateral ventricle, and the anterior horns of the lateral ventricles [3] (Figure 1).

This allows for reliable measurements of specific structures and reduced inter- and intra-sonographer variability. The correct identification of SPs is essential in the second-trimester fetal anatomic survey to investigate the morphological characteristics of the fetus and detect abnormalities or deviations from the expected growth patterns. Sonographers may struggle to obtain good SPs for various reasons, including inexperience, limited training, time limitations, and fetal movement [4], [5]. Most trainees learn to scan actual patients under the direct supervision of an expert. Although US simulators have been developed recently, trainee engagement has been limited due to competing time priorities [6]. The primary training challenge faced by all novice sonographers is not related to knowledge of anatomy or familiarity with the US machine interface. Rather, the manual navigation of the probe toward acquiring SP requires the sonographer to build a 3D map of the fetus from dynamic 2D sectional views while handling the probe. Measurements of biometric parameters and assessments of the fetal brain’s anatomy may be erroneous due to mistakes in locating the 2D scan within the 3D volume.

At present, SPs recognition represents the main focus of fetal US training. Due to the requirement to interpret variable and complex images and their spatial relationship, autonomous probe navigation toward a target plane remains challenging [7]. Our final aim is to develop a US navigation system that guides the sonographer toward obtaining SPs with reference to fetal anatomy.

In [8], we proposed a method to localize a US plane of a fetal brain directly from its 2D US image. While this is a promising result toward active guidance during fetal scanning, a few aspects still require further investigation. First, it assumes that brains from different fetuses can be mapped to the same normalized coordinate system, where each SP always has the same pose coordinates. The accuracy of this assumption has not been quantified, which limits the analysis of experimental results. Secondly, the achieved pose accuracy is still far from optimal due to a lack of variation in training data.

In this work, we expand on this work with the following contributions:

- We developed a tool for annotating poses of SPs in 3D US volumes using the Unity engine and a gaming controller.^1^ We used this tool to obtain ground truth poses of TV SPs (annotated by an obstetrician) of a publicly available fetal brain dataset. This provides a novel way of assessing the registration of fetal brain volumes from different fetuses.
- We evaluate to which extent the brains of different fetuses can be registered to the same generalized coordinate frame. While this is essential to assess training and validation data for fetal brain pose regression models, it has not been done in the previous literature on this topic [8], [9], [10].
- We evaluate different volume registration techniques and observe that manually annotating anatomical fiducials within the fetal brain is fundamental for good registration results. We demonstrate that this also contributes to the effective training of pose regression models and preserving their assumptions.
- We outperform the pose regression results obtained in [8] by improving the volume registration procedure, introducing additional data augmentation, and increasing training sets.

## II Related Work

This section presents the related work for three related but different tasks: extraction of SPs, slice-to-volume registration, and localization of SPs.

### A Extraction of Standard Planes

Previous work proposed automating the extraction of SPs from data acquired with a simplified protocol rather than assisting operators in acquiring typical freehand 2D SPs. In one of their initial works, Zhang et al. [11] developed a system based on two AdaBoost classifiers placed in cascade to automatically detect in a coarse-to-fine way early gestational sac SP. Further early studies [12], [13], [14], [15] detect key abdominal structures and landmarks in a sequence of 2D US fetal images to classify the SPs in each frame of US videos based on the presence and orientation of the landmarks using various conventional machine learning (ML) algorithms like AdaBoost, Random Forest as well as support vector machines. These methods, however, are only applicable to a subset of fetal SPs (brain and abdomen); besides, the quality of the obtained SP cannot be compared with the one achieved with typical freehand scanning.

Classification methods based on CNNs were used to detect 2D SPs because of their powerful ability to learn hierarchical representations automatically. To detect the fetal SPs, Chen et al. [16] fine-tuned a pre-trained classification CNN based on transfer learning. Baumgartner et al. [17] proposed a classification model to detect thirteen SPs with unsupervised learning and then used weakly-supervised learning (SL) based on image-level labels to locate anatomical structures in each plane. The study employed extensive data, including videos longer than those usually collected in clinical practice (roughly 30 minutes). The CNNs are fed with surrounding and additional information from each US video. To capture temporal information in 2D US, some works added to the detection of the three fetal SPs a recurrent neural network (RNN) [18].

All the methods mentioned above are effective in the detection of SP images. Still, they can only determine whether an image was captured at a SP, not where exactly it is in the corresponding 3D space. Besides, the models require a high amount of annotated data to be trained.

### B Slice-to-Volume Registration

One approach for US plane localization is to find its alignment with respect to a pre-acquired 3D volume of the same anatomy. This optimization problem is typically solved with iterative numerical methods that minimize the distance between specific landmarks or maximize intensity-based similarity metrics [19]. Unfortunately, the cost functions associated with these metrics are frequently non-convex, limiting the capture range of these registration methods. Our task differs from a classic slice-to-volume registration method, *i.e*., it does not require a previously acquired 3D volume of the same subject being scanned. Instead, we predict the pose relative to a generalized brain center, *i.e*., a stable anatomical brain point across the different, pre-aligned volumes, where training and test data belong to different subjects.

### C Localization of Standard Planes

Predicting the pose of SPs in 3D volumes can be performed without a patient-specific model and without using preoperative data. Various methods have been proposed for the localization of US planes and US probe navigation using reinforcement learning [20], [21] or external sensors [22], [23]. In the context of fetal scanning, this has been primarily approached as a classification problem, where the plane pose space is discretized into bins, and the estimation boils down to selecting one of the bins [24], [25]. In fetal magnetic resonance imaging (MRI) [26], [27] and fetal US [28], the prediction of slice locations has been previously improved with learning-based methods. General purpose learning-based methods for pose estimation approach this as a regression of a 3D translation and a 3D rotation. 3D rotations can be represented in conventional ways, such as quaternions, axis-angle, or Euler angles. Zhou et al. pointed out in [29] that, if the entire rotation space is required, these representations are sub-optimal for specific angle ranges, and proposed a new 6D representation for rotations that does not suffer from these issues. This rotation representation has been adopted for US plane localization in [8], where a regression CNN is proposed to predict the 6D pose of arbitrarily-oriented planes slicing the fetal brain US volume without the need for real ground truth data in real-time or 3D volume scans of the patient beforehand. The proposed network reliably localizes US planes within the fetal brain in phantom data and successfully generalizes pose regression for unseen fetal brains from a similar gestational age (GA) as in training. The network was tested on real fetal brain images with a GA ranging from 21 to 25 weeks. Similarly, Yeung et al. [9] proposed a CNN that takes a set of images as input and learns to compare them in pairs. The model was tested on fetal brain volumes with a GA ranging from 18 to 22 weeks. Then, in [10], the authors added an unsupervised cycle consistency using the fact that the overall displacement of a sequence of images in the 3D anatomical atlas is equal to the displacement from the first image to the last in that sequence.

## III Materials and Methods

**Fig. 2:**
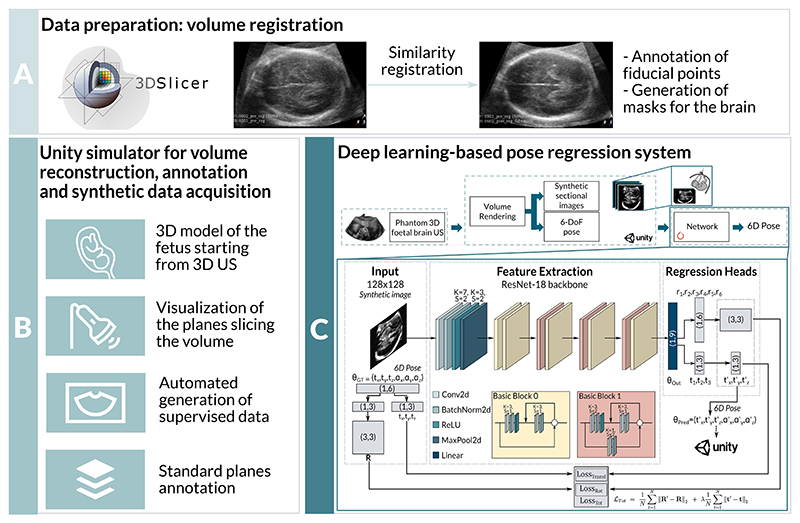
Pipeline to train and test the network. (A) The 3D fetal brain US volumes are registered in 3D Slicer using similarity registration; (B) The volumes are reconstructed into Unity, and synthetic sectional (slice) image representations are generated and saved along with their 6D pose (translation and rotation) relative to the center of the fetal brain US volume; (C) These images are fed into the network to output the estimated slice 6D pose (translation and rotation) relative to the same point.

The development of our US pose regression system has been divided into three main blocks, reported in Figure 2. First, we align 3D US volumes for the training and validation of our models. Secondly, we developed a Unity-based simulator to visualize and manually annotate SPs in 3D US volumes. This also enables the automated generation of supervised training data for our pose regression models, *i.e*., 2D synthetic images, and their ground truth 6D pose relative to the volume center. Finally, we detail our deep learning (DL)-based plane pose regression system.

### A Data Preparation

#### 1) Dataset

We utilize a selection of 6 fetal brain US volumes from a dataset of 188 volumes [30] (singleton pregnancy with no abnormal findings).^2^ The criteria for our selection is to test the generalization of a canonical frame to different fetuses within a 20–25 weeks GA range, where key anatomical landmarks can be clearly annotated for registration and evaluation. In detail, we select volumes acquired at the axial TV SP position (166 volumes); we only include volumes within the 20–25 weeks GA range (46 volumes); lastly, an experienced obstetrician excluded volumes where key landmarks (such as optic nerve) were not clearly visible along with multiple scans of the same fetus. This resulted in six real fetal brain US volumes with a GA ranging from 21 to 25 obtained from different fetuses (*f*_*i*_, with *i* = 1,…, 6). All volumes were processed to be isotropic with a voxel size of 0.5×0.5×0.5 *mm* and an average size of 249×174×155 *mm* (*coronal*×*axial*×*sagittal*, actual size of the acquired volumes).

#### 2) Volume Registration

**Fig. 3:**
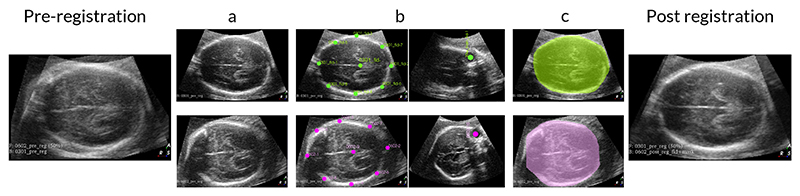
Registration procedure on real fetal brain US volumes. Workflow from pre-registration to post-registration: (a) Starting US fetal brain volumes before registration, (b) Some of the fiducial points used to achieve an initial alignment of the volumes, (c) Contour mask of the brain used to avoid overfitting on the shape of the US volume.

Even though all scans were acquired with a single protocol and in the position of the TV SP, experiments show that the anatomy within those volumes is far from well aligned and requires further registration. We tested different registration methods and obtained the best results with a fiducial-based approach utilizing 3D Slicer [31], [32]. The method is depicted in Figure 3. Starting from the initial volumes (Figure 3a), we proceed as follows:

- We annotated fiducial points to achieve an initial alignment of the volumes using the Fiducial Registration Wizard module (Figure 3b);
- Defined a contour mask of the brain using the Segment Editor module to avoid overfitting on the shape of the US volume during registration;
- Used the general registration (BRAIN) module available in 3D Slicer to register the volumes with a similarity registration phase (rigid registration + scale for a total of 7 degrees of freedom), as shown in Figure 3c. We chose the previously obtained masks as a Region of Interest (ROI) so that the registration algorithm only considers a specific image region for the registration (the fetal brain).

### B Unity Simulator for Standard Plane Annotations and Synthetic Images Generation

Starting from an open-source project,^3^ we developed our simulator using the game engine Unity [33]. The first step is rendering the volume starting from RAW, PARCHG, or Digital Imaging and Communications in Medicine (DICOM) datasets. The simulator allows the user to render the volume using three modes: Isosurface Rendering, Maximum Intensity Projection, and Direct Volume Rendering. The latter is the standard rendering mode; it uses transfer functions (1D or 2D) to determine the color and opacity while projecting rays across the dataset. Transfer functions translate density (and gradient magnitude in case of 2D) to a color and opacity. The simulator allows the user to set a custom transfer function.

- 1D Transfer Function: the density is represented on the X-axis, whereas the opacity (alpha) is on the Y-axis. The user can create a curve for opacity by density by shifting the grey alpha knots. The bottom gradient-colored panel maps color to density.
- 2D transfer function: the density is represented on the X-axis, whereas the gradient magnitude is on the Y-axis. Using the sliders, the user can define a rectangle shape, modify its size/position, and the minimum and maximum values for alpha/opacity.

#### 1) User Interface

**Fig. 4:**
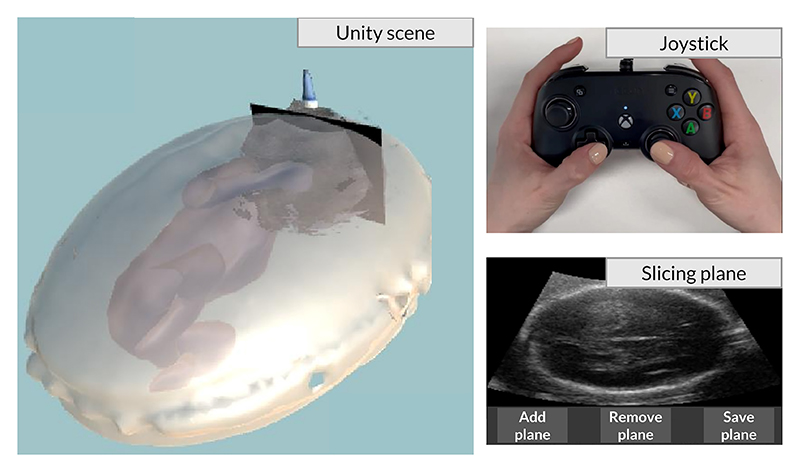
Unity simulator for volume reconstruction, SP annotations, and automatic supervised data generation. Using the suggested commands, the clinician can visualize the slicing plane in an external window while controlling the probe with the joystick. Once the desired plane is reached, it can be saved using the “Save plane” button along with its 6D pose relative to the volume center.

After loading the DICOM dataset extracted from 3D Slicer and setting the transfer function, the clinician can add a plane slicing the reconstructed 3D US volume (left-hand side of Figure 4) with an arbitrary orientation and visualize the plane in an external window (right-hand side, bottom). The plane can be controlled using a joystick (right-hand side, top), simplifying the annotation of the SPs. Hence, the clinician can modify the position and rotation of the plane using the joystick while monitoring the appearance in the external window. Once the clinicians are satisfied with the pose of the SP, they can save it using the last button in the external windows to get a picture of the slicing plane and its 6D pose relative to the volume center.

#### 2) Training and Testing Data Generation

To generate training data for our models, we generated synthetic slices by applying rotation and translation to a plane placed in the center of the volume generated with a uniform random distribution within a fixed range to avoid slices with poor overlap with the volume. The synthetic images obtained by slicing the volume were saved along with their pose with respect to the volume center (fetal brain). This provides an automated way of generating a high amount of training data with reliable ground truth labels. An obstetrician annotated the position of the TV SP by directly manipulating a slicing plane with the joystick and choosing the translation and angle sampling intervals to avoid sampling planes at the edges of the volume containing no information. The nearby planes were generated by applying small random rotations and translations (uniform distribution). Specifically, the acquisition interval between two planes was decreased from 0.1 to 0.001 for translation (Unity environment, with coordinates normalized between –1 and 1 so that the pose regression works in a fixed, normalized range, independent of the real brain size in *mm*) and from 7.9° to 1.9° for rotation compared to the acquisition of planes at random coordinates. We acquired 20699 planes with random orientation per volume and 1330 around the TV SP for a total of 22029 images for each volume.

### C Deep Learning-Based Plane Pose Regression System

We base our 6D pose regression system on the network proposed in [8] (Figure 2c). We used an 18-layer residual CNN (ResNet-18) [35] as a backbone for feature extraction with the pre-trained ImageNet weights [36]. We modified the network by re-initializing the fully connected layer based on the representation’s dimension (nine parameters) and adding a regression head to directly output the rotation and translation representations. The network receives the US image *I* (128×128) obtained by slicing the volume and its 6D pose with respect to the center of the fetal brain US volume *θ*_*GT*_ = (*t*_*x*_, *t*_*y*_, *t*_*z*_, *α*_*x*_, *α*_*y*_, *α*_*z*_). We use this information as the ground truth label for network training and validation. The CNN learns to predict the 6D pose with respect to the same point $\theta_{Pred\;}=\left(t_{x}^{\prime },t_{y}^{\prime },t_{z}^{\prime },\alpha_{x}^{\prime },\alpha_{y}^{\prime },\alpha_{z}^{\prime }\right)$. Specifically, the network first outputs a vector of nine parameters *θ*_*Out*_ = (*t*_1_, *t*_2_, *t*_3_, *r*_1_,…, *r*_6_); the first three are used for the translation and the last six for the rotation. Then, *r*_1_,…, *r*_6_ are used internally by our CNN to reconstruct the rotation matrix **R**′ in the forward pass. Differently from [8], we also perform image intensity augmentation. More specifically, we change brightness, contrast, and saturation. The detailed parameters of this augmentation are described in the next section.

## IV Experiments And Results

In this section, we report the results of three main experiments. First, in Section IV-A, we assess three different US volume registration methods and investigate their qualitative and quantitative impact in defining a generalized inter-patient coordinate frame for the fetal brain (Sections IV-A1 and IV-A2). Besides, we illustrate the effect of these registration methods when training US plane pose regression networks using a fetal US volume of a single 23-week fetus to train the network and fetuses with a GA ranging from 21 to 25 weeks for testing (Section IV-B). In our second experiment, in Section IV-C, we investigate how consistent the manual annotations of the TV SPs are both in terms of quality (Section IV-C1) and variability (Section IV-C2) and assess their role in evaluating the quality of volume registration and pose regression. Lastly, Section IV-D reports final pose regression results with Leave One Out Cross-Validation (LOOCV) when training data has the best registration alignment available and intensity data augmentations are performed.

### A Volume Registration

**Fig. 5:**
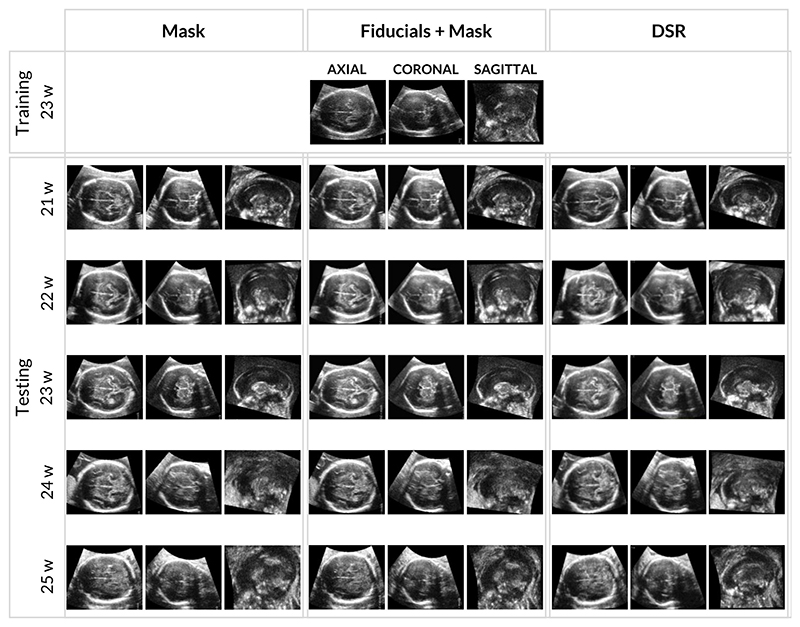
Axial, coronal, and sagittal views of the middle slices of each volume after registration on real fetal brain US volumes. First column: results obtained by aligning the volumes using a mask to contour the brain used to avoid overfitting on the shape of the US volume and then applying the automatic similarity registration (rigid registration + scale for a total of 7 degrees of freedom) provided by 3D Slicer. Second column: results obtained by aligning the volumes with the automatic registration using the fiducial points annotated by the obstetrician, followed by similarity registration using the mask that contours the brain. Third column: results obtained by aligning the volumes with DSR registration [34].

Before training the pose regression models, we require a set of well-aligned 3D US volumes to generate training and validation data. Figure 5 reports the axial, coronal, and sagittal views of the middle slice for each US volume after aligning them with three different volume registration methods. The first column shows the results obtained using direct similarity registration on raw US volume data (rigid registration + scale for a total of 7 degrees of freedom) performed only on a manually annotated mask, a ROI that contours the fetal brain; this registration uses the Mattes Mutual Information (MMI) image comparison cost metric during fitting. We provide an identity transformation for initialization. The second column shows the results obtained with a point-based registration with fiducial points annotated by an obstetrician, followed by similarity registration with a fetal brain mask. The third column shows the results obtained with a state-of-the-art intensity registration approach called Direct Simultaneous Registration (DSR). Since the method is iterative, we initialized it with the fiducial approach and performed a mono-modal rigid registration of multiple volumes [34].

Table I reports the evaluation of the different registration methods and their effects on US plane pose regression. We performed two different types of evaluation.

#### 1) Registration Error

Registration accuracy is usually evaluated by identifying matching pairs of landmarks annotated by the clinician in the ROI. We report the error between the volumes prior to registration with a GA ranging from 21 to 25 weeks and the one used for training (23 weeks); besides, we report the Root Mean Square (RMS) error between the landmarks in *mm* and an intensity-based RMS score for the same volumes for the three registration methods;

#### 2) Obstetrician’s Evaluation

We asked the obstetrician to evaluate the registration outcome for the three registration methods, shown in Figure 5, by assigning a score between 1 (bad) and 5 (good), without taking into account the quality of the volumes;

### B Registration Impact on CNN Results

We measure the errors of our pose regression CNN according to the different “ground truths” resulting from different registration methods. We evaluate to which extent the different volume registration methods affect the perceived US plane pose regression results.

#### Implementation Details

Our framework is implemented in PyTorch and trained using a single Tesla^®^ A100-SXM4-40GB hosted on the Computer Science network at University College London. The network was trained for 50 epochs with a batch size of *K* = 64 using Adam optimizer, with a learning rate of 0.0001 and exponential decay rates *β*_1_ and *β*_2_ of 0.9 and 0.999, respectively. We choose the best model weights considering mean square error (MSE) obtained on the validation set (20% of the training set).

#### Experiments

**Fig. 6:**
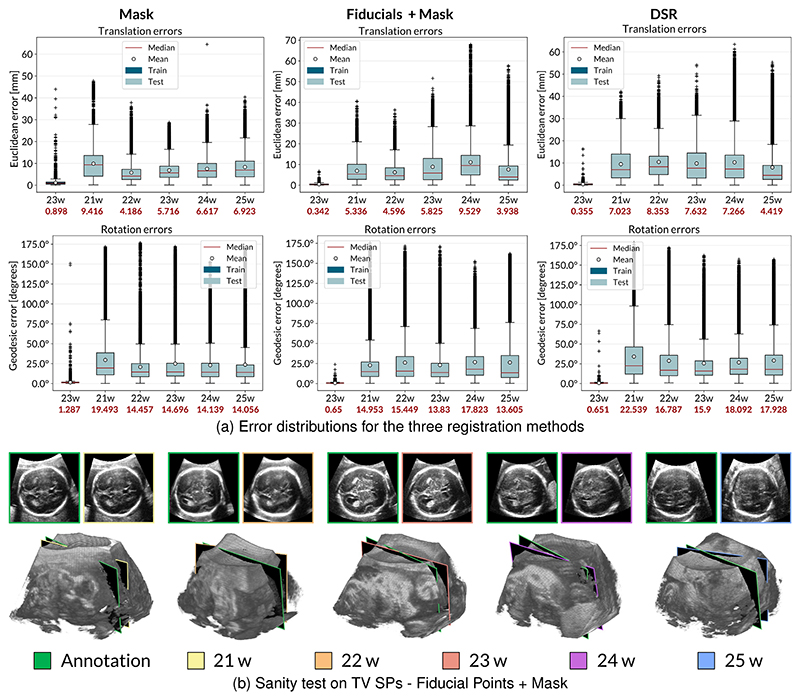
CNN results for the different registration methods, trained on a single 23w volume. (a) Translation and rotation error distributions for both planes acquired at random coordinates and planes acquired around the annotated TV SP to analyze the generalization capabilities of the network with the three registration methods. (b) TV SP prediction performed by the regression CNN. The green and the colored boxes indicate the ground truths and the predictions, respectively. An obstetrician within the Unity environment manually annotated the ground truth poses of the TV SPs.

The network was trained on phantom data and fine-tuned on real ones [8]. Specifically, we fine-tuned the network on planes extracted from a fetal brain US volume with a GA of 23 weeks (*f*_1_, 22029 images). We tested it on planes from five volumes obtained with a single acquisition of different fetuses (*f*_2_,…, *f*_6_) ranging from a GA of 21 to 25 weeks to understand how well the model generalizes over different shapes and sizes. Images were resized to 128×128, preserving the same aspect ratio, and cropped and centered to avoid visible sharp edges that could cause overfitting. We augmented the training set by randomly changing the images’ brightness, contrast, and saturation to a value between 0 and 1. To this aim, we used the torchvision.transforms.ColorJitter class that is available in Pytorch for transforming and augmenting images. We employed the Euclidean distance between the two planes to evaluate the translation results, reported in *mm*. For rotation, we display errors as the geodesic distance to ground truth in *degrees*, more suitable for the geometric interpretation of the distance between two 3D rotations and defined as $\;Error{\;}_{Rotation\;}=\arccos\left(\left(R_{00}^{\prime \prime }+R_{11}^{\prime \prime }+R_{22}^{\prime \prime }-1\right)/2\right)$, where **R**^″^ = **R**^′−**1**^. Table I reports the median errors for translation and rotation obtained on the testing volumes for the three registration methods. Figure 6 reports the translation and rotation error distributions for fetal brain US volumes ranging from a GA of 21 weeks to 25 weeks to analyze the generalization capability of the network in the three registration methods. Besides, we performed a sanity test using the manually annotated TV SPs for the registration using the fiducial points and the mask. The sectional images were saved and fed into the network to estimate their pose. We plotted the two planes within the volume in Unity to visually evaluate the distance between the annotated TV SPs and the predicted ones. The predicted planes were also fed into a pre-trained^4^ SonoNet, a CNN that can automatically detect 13 fetal standard views in freehand 2D US data [17], in its Pytorch implementation. Figure 6 reports the annotated TV SPs (green edges) and the ones having the pose predicted by the regression network in their sectional view and within the volumes.

### C Standard Planes Annotations

An obstetrician annotated the SPs for all the 3D US fetal brain volumes previously registered using the Unity simulator detailed above.

#### 1) Quality of Annotations

**Fig. 7:**
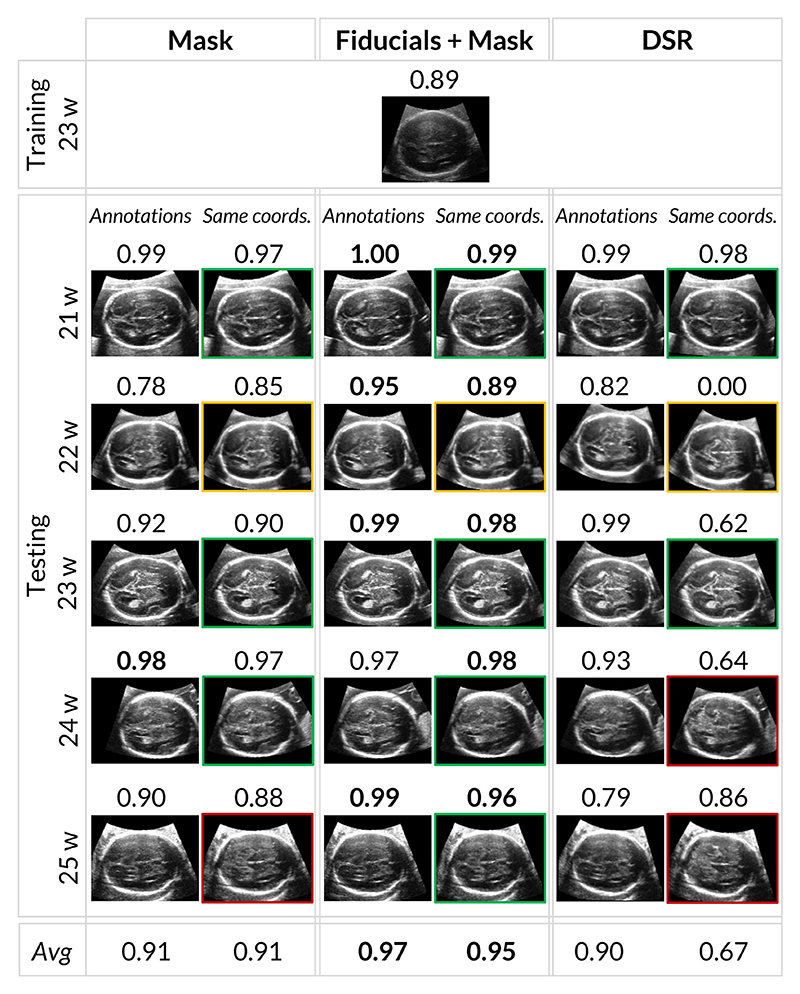
Evaluation of different registration methods according to SP alignment. For each method, the first column denotes manually annotated SPs for each volume, and the second denotes a slice at fixed coordinates where the SP is expected, assuming the same coordinates as the reference volume (23 w). Colors denote whether the SP follows clinical guidelines [2]. Green box: the SP is of good quality; Orange box: the SP does not contain the CSP, which should be visible; Red box: the SP contains the cerebellum, which should not be visible. The numbers denote SonoNet confidence in correctly identifying the correct SP.

To evaluate the quality of the annotated TV SPs, we use SonoNet in its PyTorch implementation. We report the annotated TV SPs for the various volumes and the registration methods in Figure 7. SonoNet was able to classify all the annotated SPs as TV SPs, the brain view at the posterior horn of the ventricle. We then applied the coordinates of the TV SP annotated on the training volume (23 w) to the other planes. The synthetic images obtained for the different volumes were fed again into SonoNet to understand if the network could still recognize the planes as standard views. All the planes were recognized as TV SPs, except for the volume with a GA of 25 weeks obtained using the automatic registration. The confidence of the classification (value between 0 and 1) is reported on top of each image in Figure 7. For each registration approach, the first column shows the TV SPs obtained from the annotations. In contrast, the second column shows the TV SPs obtained by using the coordinates of the TV SP annotated on the training volume. An obstetrician evaluated the appearance of SPs shown in the second column to ensure that the obtained planes follow the relevant clinical guidelines [2]. In this context, some of the planes are of poor quality due to the absence of the CSP, shown in Figure 1, and are marked in orange; other planes display the cerebellum which should not be visible and are marked in red. At the bottom, we report the average score, including the training volume (23 w).

#### 2) Variability in SPs Annotations

We evaluate the variability in annotations by reporting the standard deviation of the poses of the TV SPs annotated by the obstetrician in the various volumes. The internal variance of the entire set provides the quality of the data. We report the variance in translation and rotation of the annotated TV SPs for each registration approach. For translation, we computed the coordinates of the centroid *c*_*transl*_ = *x*_*c*_, *y*_*c*_, *z*_*c*_ for each group made of the training volume and the five testing volumes (*i* = 1,…, 6) as the mean on of three coordinates:

(1) $$x_{c}=\frac{\sum_{i=1}^{6}x_{i}}{6},y_{c}=\frac{\sum_{i=1}^{6}y_{i}}{6},z_{c}=\frac{\sum_{i=1}^{6}z_{i}}{6}$$

Then, we computed the Euclidean distance between each TV SP and the centroid:

(2) $$d_{i,\;transl\;}=\sqrt{{\left(x_{i}-x_{c}\right)}^{2}+{\left(y_{i}-y_{c}\right)}^{2}+{\left(z_{i}-z_{c}\right)}^{2}}$$

Lastly, we computed the RMS of these distances:

(3) $$RMS_{transl\;}=\sqrt{\frac{1}{6}\sum_{i=1}^{6}d_{i,\;transl\;}^{2}}$$

We computed the Chordal L2-averaging of the rotation matrices for rotation, following the approach presented in [37]. This is achieved by finding the rotation *R*_*c*_ that minimizes the cost $\sum_{(i,j)\in N}{\left\|R_{ij}R_{i}-R_{j}\right\|}_{F}^{2}$. The above model can be solved without enforcing the orthogonality constraint as a least squares problem through vectorization and singular value decomposition. After that, all the orthogonal constraints are enforced by finding the nearest orthogonal matrices through polar decomposition. Then, we computed the geodesic distance between the rotation matrix of each TV SP and the average rotation matrix (angle of residual rotation):

(4) $$d_{i,rot}\left(R_{i},R_{c}\right)=d_{i,rot}\left(R_{i}R_{c}^{T},I\right)={\left\|log\left(R_{i}R_{c}^{T}\right)\right\|}_{2}$$

where the norm is the Euclidean norm in ℝ^3^. The angular distance function *d*_*i,rot*_(*R*_*i*_, *R*_*c*_) is equal to the rotation angle $∠\left(R_{i}R_{c}^{T}\right)$. Starting from the quaternion representations, it is possible to easily compute the angular distance between two rotations. If *r*_*i*_ and *r*_*c*_ are quaternion representations of *R*_*i*_ and *R*_*c*_ respectively, and *θ* = *d*_*i,rot*_(*R*_*i*_, *R*_*c*_), then *θ* = 2*arccos(*|*s*|), where $(s,v)=r_{c}^{-1}\cdot r_{i}$. The absolute value sign in *s* is required to account for the sign ambiguity in the quaternion representation of the rotation $R_{i}^{T}R_{c}$. The positive sign is chosen so that the angle *θ* lies in the range 0 ≤ *θ* ≤ *π*, as required. Hence, the distance *d*_*i,rot*_(*R*_*i*_, *R*_*c*_) is equal to the angle *θ* belonging to the rotation $R_{i}R_{c}^{T}$. As before, we computed the root mean square of the distances:

(5) $$RMS_{rot\;}=\sqrt{\frac{1}{6}\sum_{i=1}^{6}d_{i,rot}{\left(R_{i},R_{c}\right)}^{2}}$$

**Fig. 8:**
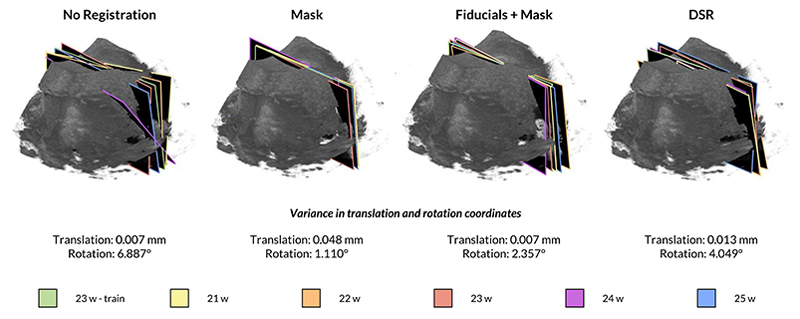
TV SPs annotated by the obstetrician for the three registration methods and variance in translation and rotation coordinates.

The results are reported in Figure 8 along with the appearance of the TV SPs annotated by the obstetrician for the three registration methods.

### D Ablation Study on the Pose Regression CNN

We performed an ablation study on the volumes registered using the combination of fiducial points and masks. First, we present the results for the US plane pose regression with and without data augmentation on the training set. Then, we extend the training set and combine data augmentation with LOOCV.

#### 1) Data Augmentation

Data augmentation artificially boosts the size and variance of the training dataset by including transformed copies of the training examples. This is especially useful in medical imaging, where data augmentation is applied to expand training data, address the class imbalance and improve model generalization. To understand to which extent data augmentation could benefit our training, and increase the generalization over different shapes and sizes, we augmented the training set, as detailed above.

#### 2) Leave-One-Out Cross-Validation

The localization errors increase when the training distribution differs from the testing one. A better training and testing data distribution design can accurately reflect the model’s performance during application. Hence, to estimate the performance of our algorithm in making predictions on data not used to train the model, we performed LOOCV experiments using the volumes with a GA ranging from 21 to 25 weeks, including the one originally used for training (23 weeks), for a total of six volumes (*N* = 6). LOOCV is a special case of *k*-fold cross-validation with *k* = *N*, the number of volumes. LOOCV involves one fold per volume, *i.e*., each volume plays the role of the validation set. The (*N* − 1) volumes play the role of the training set. With least-squares linear, a single model performance cost is the same as a single model. The average error on the test set is calculated by fitting on the volumes not used in training and gives us an idea of how well the model will perform on data it has not previously seen. We then calculate the error on the test set *Test Err*_*avg*_ to be the average of all the errors on the six test sets *Test Err*_*i*_:

(6) $$\;Test\;Err{\;}_{avg\;}=\frac{1}{N}\sum_{i=1}^{N}\;Test\;Err\;r_{i}$$

**Fig. 9:**
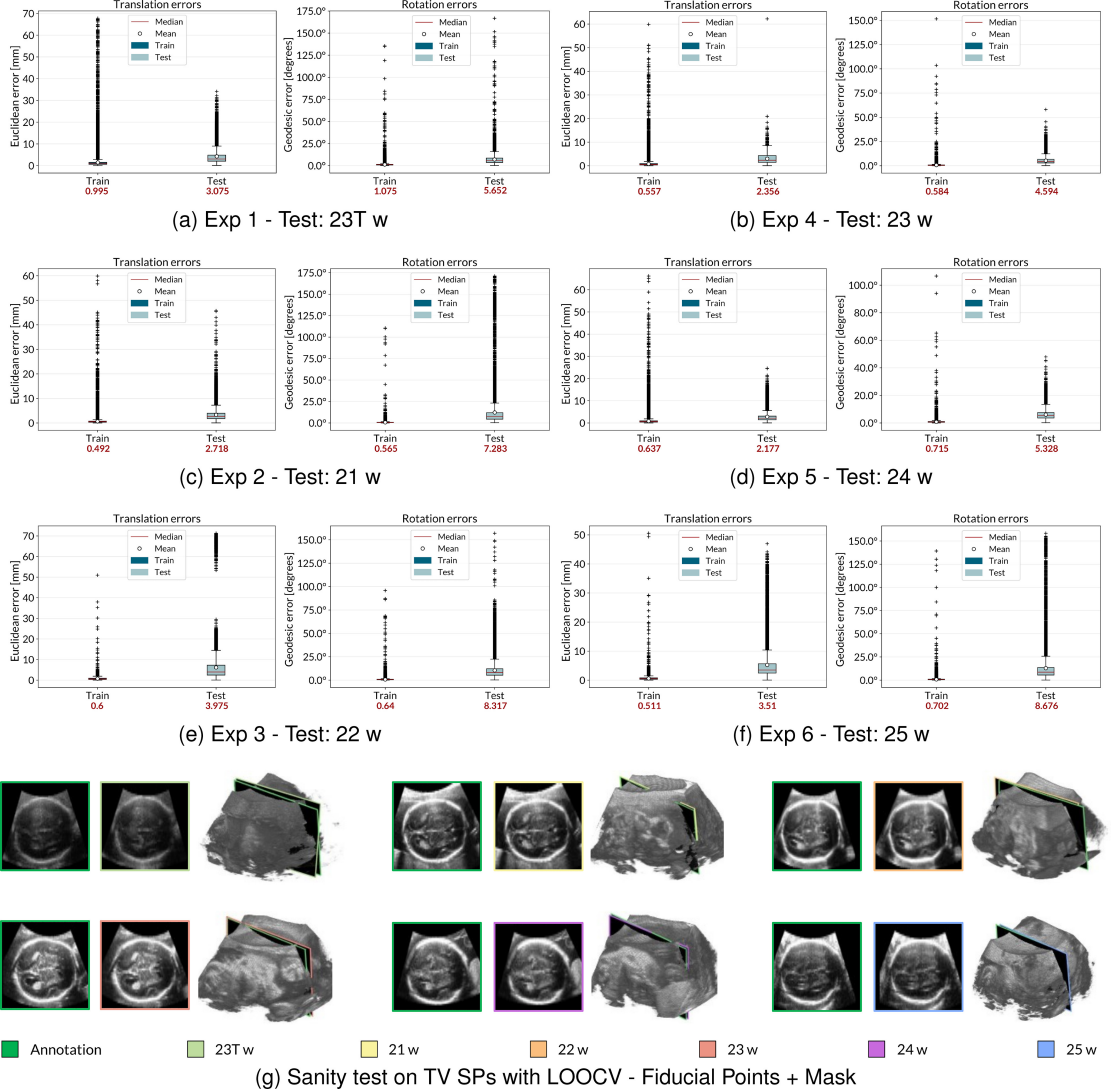
Results obtained for the experiments performed with the LOOCV. (a-f) Translation and rotation error distributions for the LOOCV experiments for the six cases. (g) TV SP prediction performed by the regression CNNs. The green and the colored boxes indicate the ground truths and the predictions, respectively. An obstetrician within the Unity environment manually annotated the ground truth poses of the TV SPs. 23T indicates the volume having a GA of 23 weeks used for reference in the registration and training in the previous experiments. The consistency between the annotated and predicted SPs is demonstrated by the fact that the planes show the same anatomical structures.

Figures 9a-f show the translation and rotation error distributions for the LOOCV experiments for the different trained models. Figure 9g reports the results for the sanity test performed using the manually annotated TV SPs, as previously detailed in Section IV-B. Table II reports the median, mean ± standard deviation, maximum, and minimum errors and the average error with and without data augmentation (Equation (6)).

## V Discussion

First, we note that even though all the utilized volumes in this study were acquired with the same protocol to capture the TV SP plane, this does not mean the anatomies in different volumes are well aligned. This is evident from pre/post-registration results in Table I and Figure 8. The reasons can include different fetus positions in the womb, different fetus brain sizes, operator variability, and US machine settings.

Our volume registration results in Table I indicate that Fiducials+Mask provides the best alignment in terms of fiducial errors. We argue that the alignment of such anatomical structures is of utmost importance in our application context. The results in Figure 7 further support that Fiducials+Mask provides the best alignment of clinically relevant anatomy by assessing the expected location of the TV SP plane across different fetuses, showing higher consistency than other methods. On the other hand, DSR is the best performing in terms of intensity errors (Table I). However, this may indicate overfitting since the same anatomical landmarks in different scans of different fetuses do not necessarily have the same intensity values. Regarding the obstetrician evaluation, DSR slightly outperforms Fiducials+Mask. This metric reflects a qualitative skull alignment in axial/coronal/sagittal brain views. Finally, the results from Figure 8 suggest that annotated TV SPs on each fetus are the most consistent in translation for Fiducials+Mask and the most consistent in rotation for Mask. While rotation and translation alignment favor different methods, we observe that the best translation alignment of TV SPs (Fiducials+Mask) overlaps with the best alignment of anatomical features overall. In summary, the metrics most directly linked to the correct alignment of structures inside the brain (fiducial errors and TV SP image assessment) suggest that Fiducials+Mask provides the best registration results. With this in mind, we believe this method provides the most appropriate registration to validate the generalization of brain structure locations across different fetuses and use it to train and test our CNN. After this alignment, a set of SP from different fetuses, manually annotated by an obstetrician, has a variance of 0.007 *mm* and 2.357° in translation and rotation, respectively. These values are indicative of the ground truth uncertainty and, therefore, provide an estimated lower bound for our CNN pose regression accuracy.

The results in Figure 6a demonstrate that different volume registrations significantly impact the interpretation of regression CNN results, simultaneously affecting the trained model’s perceived quality and the ground truth. While this effect was mentioned in [8] as a study limitation, here we quantify its impact. We note that better CNN results do not necessarily mean better registration quality due to variations in the ground truth. We present these results to highlight that CNN results alone are insufficient to assess registration quality and only indicate how well the training and test data fit together.

After establishing Fiducials+Mask as the most trustworthy ground truth alignment, we fully assess our CNN with our LOOCV study (Figure 9). Table II shows that we outperform the pose regression results obtained in [8] due to many factors. These include the more rigorous volume registration process, larger training sets that include various GAs, additional image intensity data augmentation, and removal of a fully connected layer before final regression. The proposed CNN successfully generalizes pose regression to an unseen fetal brain. Specifically, data augmentation decreased median errors by 22.86% and 10.88% for translation and rotation, respectively. Besides, by extending the training sets, we could further decrease the median errors and obtain a better generalization. Our model is designed to be size invariant by performing pose regression in normalized coordinates with respect to the brain limits. However, GA does not only affect size but also shape. Indeed, including different GAs covers a wide range of shapes and sizes, enabling us to understand our model’s current generalizability and limitations.

Despite our promising results relative to the existing literature [8], [9], [10], our regression network still produces outlier predictions with large errors. This is to be expected, given that we perform estimations based on single US scans, which can be noisy. Our median results still suggest that the large majority of predictions has a relatively small error; hence, this is a promising backbone for any future work aiming at pose regression from continuous US video where sparse erroneous predictions could be filtered with temporal models (*e.g*., sliding window filtering/regularization, LSTMs, transformers).

## VI Conclusion

In the context of fetal US plane pose regression, our study highlights the need for an application-specific registration methodology to align training and test US volumes from different fetuses. The algorithm design and its evaluation should focus on the explicit alignment of anatomical features rather than volume intensities.

The obtained registration results provide promising evidence that assuming generalized coordinates for the fetal brain is a valid assumption within a small tolerance, especially in the context of anatomies present in the TV SP. However, our data only includes 6 volumes, and further analysis will require detailed annotation of additional volumes. Besides, in our experiments, we make use of inter-patient volume-to-volume US registration, a domain with limited tailored algorithms. Intensity-based approaches such as DSR or accumulated pairwise estimates (APE) [38] are not optimal for interpatient registration. Potential improvements could be achieved with landmark-based registration approaches [39], [40], [41]. However, these are not off-the-shelf applicable to our interfetus registration problem; thus, adapting them requires further work and additional training data.

We fully re-assess the results of our CNN pose regression network for localizing fetal brain US scans in light of the improved registration methodology. We can estimate plane poses within the brain with a median translation error of 2.97 *mm* and a median rotation error of 6.63°. These results are promising, given that we are localizing images of a previously unseen fetus from a single frame. However, there are still outlier predictions with large errors (refer to maximum errors in Table II). We believe the key to address this challenge is to move away from single-frame estimation and take temporal cues into account.

This work could potentially be generalized to other anatomical regions of the fetus, such as the abdomen; however, the definition of a generalized reference frame would still be challenging due to increased deformations.

We will also assess the potential of the current work toward active guidance of sonographers during SPs acquisition for fetal biometry. This could include automated feedback signals to guide a novice from an arbitrary US plane toward the target SP.

## Figures and Tables

**Table 1:** EVALUATION OF THE DIFFERENT REGISTRATION METHODS. WE REPORT THE ROOT MEAN SQUARE (RMS) ERROR FOR MANUALLY ANNOTATED FIDUCIALS AND THE RMS ERROR OF RAW VOLUME INTENSITIES, BOTH WITH RESPECT TO A REFERENCE 23 WEEK VOLUME. WE ALSO PROVIDE THE OBSTETRICIAN’S ASSESSMENT OF SKULL ALIGNMENT BASED ON AXIAL, CORONAL, AND SAGITTAL PLANES ON A 1 (BAD) TO 5 (GOOD) SCALE

	Evaluation Metric	No registration	Registration		
			Mask	Fid + Mask	DSR
	Fiducials Errors (RMS [*mm*])	6.02	5.82	**5.80**	6.51
21w	Volume Intensity Errors (RMS)	*62.17*	48.63	47.73	**44.04**
	Obstetrician (1-5)		4	**5**	**5**
	Fiducials Errors (RMS [*mm*])	2.53	2.56	**2.52**	5.08
22 w	Volume Intensity Errors (RMS)	*53.73*	39.04	40.08	**37.53**
	Obstetrician d (1-5)		4	**5**	**5**
	Fiducials Errors (RMS [*mm*])	3.34	3.86	**3.32**	6.07
23 w	Volume Intensity Errors (RMS)	*45.27*	38.51	39.09	**37.05**
	Obstetrician d (1-5)		4	**5**	**5**
	Fiducials Errors (RMS [*mm*])	3.45	3.74	**3.00**	5.36
24 w	Volume Intensity Errors (RMS)	*49.53*	43.99	41.16	**40.08**
	Obstetrician d (1-5)		3	3.5	**4**
	Fiducials Errors (RMS [*mm*])	2.19	3.89	**1.68**	5.00
25 w	Volume Intensity Errors (RMS)	*56.14*	45.19	41.43	**34.12**
	Obstetrician (1-5)			3.5	
	Fiducials Errors (RMS [*mm*])	3.51	3.97	3.27	5.60
Avg	Volume Intensity Errors (RMS)	*53.37*	43.08	41.89	**38.56**
	Obstetrician d (1-5)		3.6	4.4	**4.6**

**Table II:** TRANSLATION AND ROTATION ERRORS OF OUR METHOD FOR THE LOOCV EXPERIMENTS ON INCLUDING DATA AUGMENTATION. NORM: EUCLIDEAN DISTANCE, GE: GEODESIC ERROR, DA: DATA AUGMENTATION, SD: STANDARD DEVIATION, 23T INDICATES THE VOLUME HAVING A GA OF 23 WEEKS USED FOR REFERENCE IN THE REGISTRATION AND TRAINING IN THE PREVIOUS EXPERIMENTS

Test Volume	DA	Translation - Norm [mm]					Rotation - GE [deg]		
	Yes	*Median*	*Mean±SD*	*Min*	*Max*	*Median*	*Mean±SD*	*Min*	*Max*
23T w	Yes	3.07	4.24±3.71	0.09	34.09	5.65	6.97±6.41	0.13	166.77
21 w	Yes	2.72	3.30±2.67	0.04	45.75	7.28	12.18±19.03	0.29	171.10
22 w	Yes	2.18	2.79±2.36	0.03	24.61	5.33	6.02±3.65	0.18	47.97
23 w	Yes	2.36	2.96±2.02	0.03	62.22	4.59	5.27±3.48	0.09	58.03
24 w	Yes	3.97	6.17±8.09	0.05	71.29	8.31	10.45±9.48	0.23	156.98
25 w	Yes	3.51	5.23±5.40	0.03	46.96	8.67	12.91±16.27	0.09	158.32
*Test Err_avg_*	No	3.85	4.96±3.98	0.06	42.12	7.44	7.05±8.23	0.19	120.41
	Yes	2.97	4.11 ±4.04	0.05	47.48	6.63	8.96±9.72	0.16	126.52

## Footnotes

1 FetalUltrasoundSimulator-Unity - GitHub.

2 Refer to www.datavers.nl for details.

3 UnityVolumeRendering.

4
https://github.com/baumgach/SonoNet-weights
